# Double Balloon Cervical Ripening Catheter for Control of Massive Hemorrhage in a Cervical Ectopic Pregnancy

**DOI:** 10.1155/2017/9396075

**Published:** 2017-02-02

**Authors:** Nabila Zambrano, James Reilly, Michael Moretti, Nisha Lakhi

**Affiliations:** ^1^Department of Obstetrics and Gynecology, Richmond University Medical Center, 355 Bard Avenue, Staten Island, NY 10310, USA; ^2^Department of Obstetrics and Gynecology, New York Medical College, 40 Sunshine Cottage Rd, Valhalla, NY 10595, USA

## Abstract

Cervical pregnancy can be complicated by perfuse vaginal bleeding. Mechanical compression directed at tamponing the cervical vessels can control hemostasis. There are several types of balloon catheters that have been described for cervical compression. However use of a double balloon catheter is a novel approach for cervical tamponade, as one balloon is positioned below the external cervical os and the second balloon is situated above in the internal cervical os. This compresses the cervix from internal os to external os between the two balloons, forming a “cervical sandwich.” We describe this method of cervical tamponade using a silicone double balloon cervical ripening catheter that rapidly controlled hemorrhage in a patient that failed conservative management with methotrexate.

## 1. Introduction

Cervical pregnancy (CP) is a rare type of ectopic pregnancy characterized by implantation of a fertilized ovum in the endocervical canal. CP accounts for less than 1% of all ectopic pregnancies [1–3]. Risk factors include endometrial damage after curettage or chronic endometritis, leiomyoma, intrauterine devices, and in vitro fertilization [2, 4, 5]. This type of ectopic pregnancy is highly dangerous because massive hemorrhage can ensue secondary to bleeding from the cervical vessels. Early detection has been improved by ultrasonography. However, even with advanced diagnostic modalities and treatment options, CP remains a life-threatening condition.

During the last decade, in an effort to avoid hysterectomy and maintain fertility, a more conservative therapeutic approach has been advocated. This includes chemotherapy with methotrexate, intra-amniotic feticide, dilation and curettage, uterine tamponade, and uterine artery embolization [5, 6]. Usually more than one modality of treatment is necessary [2, 7]. We describe a novel method of cervical tamponade using a silicone double balloon cervical ripening catheter (Cook® Cervical Ripening Balloon, Cook Medical, Bloomington, Indiana) in a patient that presented with acute hemorrhage after conservative management with systemic chemotherapy.

## 2. Case

A 31-year-old gravida 2 para 0 at 7 weeks and 3 days presented to the emergency room with complaints of vaginal bleeding and abdominal cramping that began earlier that morning. On examination, her abdomen was nontender without rebound or guarding. No active bleeding was noted vaginally. Her serum beta human chorionic gonadotrophin (*β*-hCG) was 18,645 mIU/L.

Transvaginal ultrasound revealed a 22 mm gestational sac implanted within the cervical canal with a fetal pole but no fetal cardiac activity. Decision was made to admit patient for systemic multidose methotrexate regimen for conservative management of a cervical pregnancy. Uterine artery embolization was considered but concern arose about long term effects on the patient's future fertility.

Methotrexate 85 mg (1 mg/kg) was administered on days 1, 3, 5, and 7. Rescue leucovorin 8.5 mg (0.1 mg/kg) was administered on days 2, 4, 6, and 8. Her *β*-hCG levels on days 1, 3, 5, and 7 were 18,645 IU/mL, 15,527 IU/mL, 15,099 IU/mL, 12,718 IU/mL, and 10,222 IU/mL, respectively. On hospital day 7, repeat transvaginal ultrasonography showed a collapsing gestational sac with no visible yolk sac or fetal pole. The patient experienced no further vaginal bleeding during admission. The patient was discharged home with plan of having weekly *β*-hCG measured until levels declined to zero.

**Figure 1:**
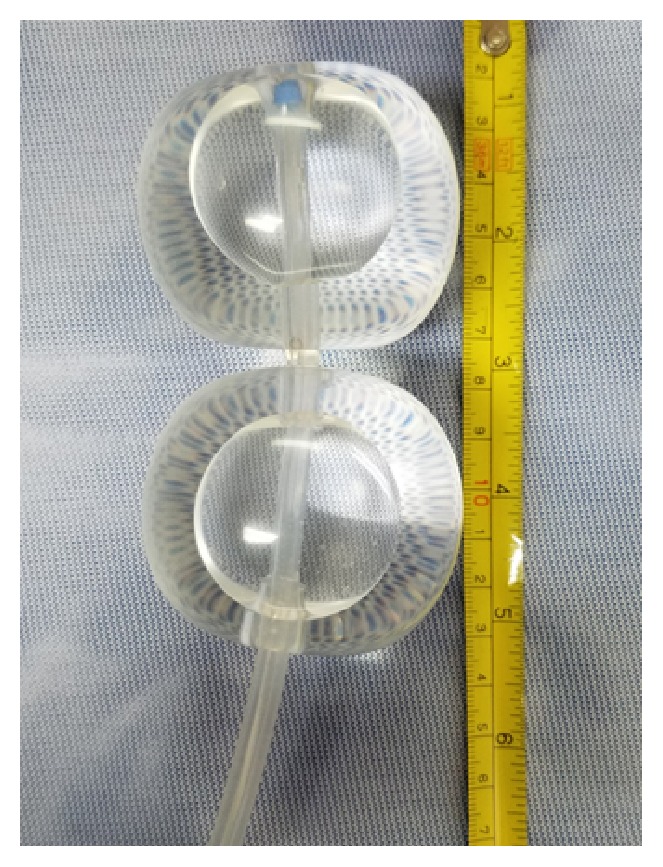
Cook Balloon Inflated with 80 mL saline in the uterine balloon and 60 mL saline in vaginal balloon.

**Figure 2:**
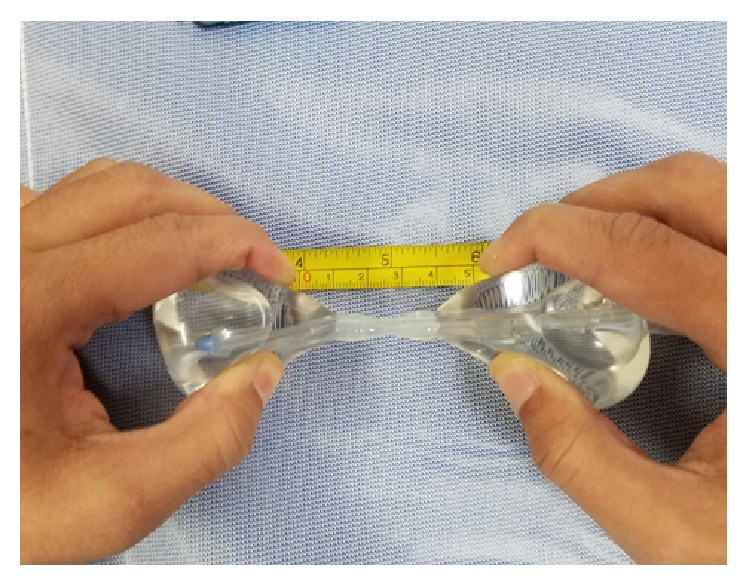
Distance between the two balloons can be stretched approximately 5 cm.

**Figure 3:**
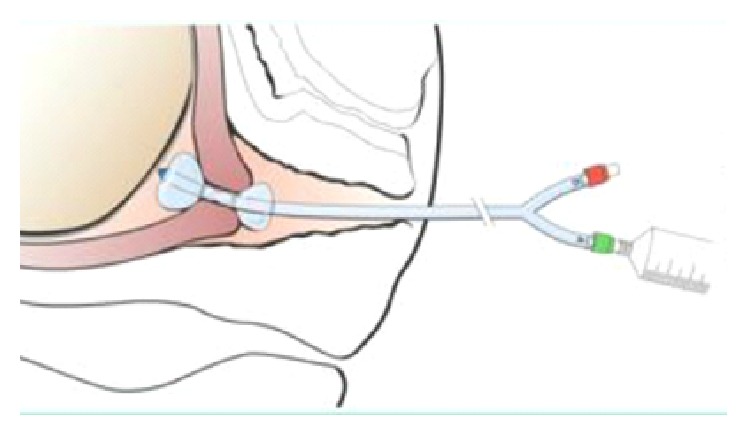
Placement of balloons in uterus and vagina (picture from package insert).

**Figure 4:**
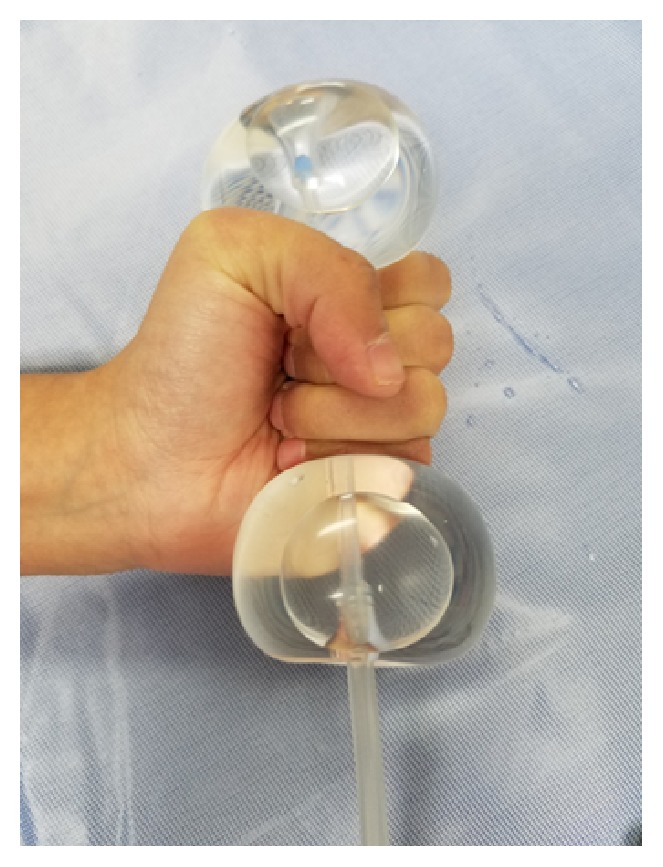
Illustration of “sandwich effect” of the two inflated balloons. Note that space between the balloons is stretchable and can accommodate the entire cervix.

The patient returned to the emergency room two days after discharge with heavy vaginal bleeding. On examination, approximately 300 mL of blood was cleared from the vaginal vault. The vaginal vault was packed with gauze in an attempt to tamponade bleeding so that patient could be safely transported to the operating room for an examination under anesthesia. At the time of the patient's presentation to emergency room, interventional radiology was not readily available to perform uterine artery embolization. After removal of vaginal packing, approximately 500 mL of additional vaginal bleeding was noted. Dilation and suction curettage was performed in order to excise the remaining trophoblast tissue that was readily visualized at the cervical os. After curettage, the patient continued to actively hemorrhage. A silicone double balloon cervical ripening catheter (Cook Cervical Ripening Balloon) was introduced into the cervical canal (Figures 1 and 2). 80 mL of normal saline was inserted in the uterine valve and 60 mL of normal saline inserted in was introduced into the vaginal valve. Upon insufflation, the cervix was compressed between the two balloons of the catheter and hemostasis was rapidly achieved (Figures 3 and 4). Positioning of the Cook Balloon was ascertained by visual inspection and by gentle traction. Gentle traction insured that the uterine balloon of the Cook catheter was securely positioned above the internal os after insufflation. Once the uterine balloon was determined to be in the appropriate place, the vaginal valve was insufflated under direct visualization. Due to the acuity and urgency to stabilize the patient and to minimize bleeding, imaging to document placement could not be performed during the procedure.

The patient was observed overnight and reexamined the following morning. No further bleeding was noted and the patient remained hemodynamically stable. The compression balloon was removed without difficulty after 12 hours. The patient received two doses of tranexamic acid (1000 mg) approximately 18 hours apart after removal of Cook Balloon. The patient was discharged the following day in stable condition. *β*-hCG repeated 24 days after discharge from the hospital was 2 IU/mL.

## 3. Discussion

**Figure 5:**
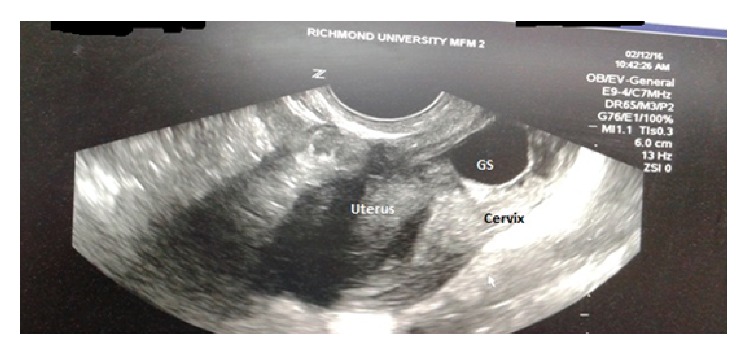
Empty uterus with gestational sac (GS) in cervix.

**Figure 6:**
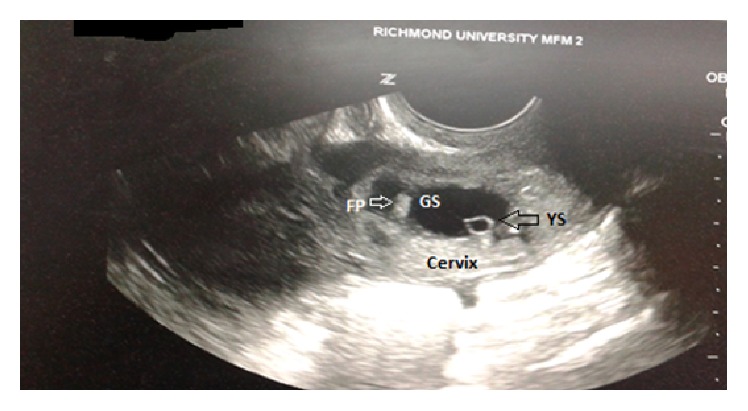
Gestational sac with yolk sac and fetal pole within the cervix.

**Figure 7:**
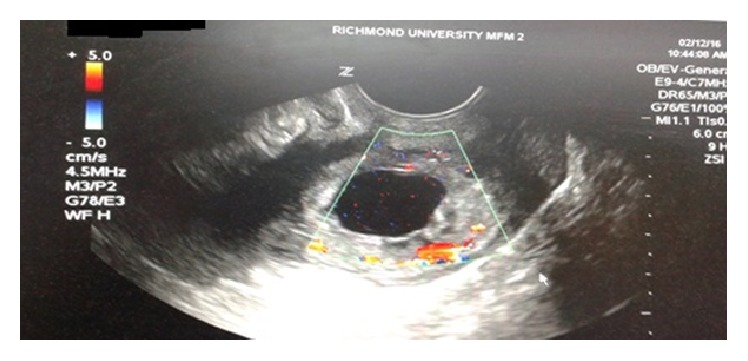
Vascularization around the gestational sac demonstrated by Doppler color flow.

The early diagnosis of cervical ectopic pregnancy in our patient was made by confirming the following ultrasound criteria: (1) an empty uterine cavity above the level of the internal cervical os (Figure 5), (2) barrel-shaped cervix (Figure 6), (3) gestational sac located below the level of the uterine arteries, and (4) vascularization around the gestational sac demonstrated by Doppler color flow ultrasonography (Figure 7) [7, 8]. Cervical pregnancy can be distinguished from an imminent aborting pregnancy within the cervix by the absence of the “sliding sign” (elicited when mechanical pressure from the ultrasound transducer causes the gestational sac to slide against the endocervical canal in case of imminent abortion but not in an implanted CP) [7].

Although there are no definitive guidelines for the management of cervical pregnancies, a variety of modalities have been proposed [4]. Approaches include curettage combined with Foley balloon tamponade, methotrexate with/without potassium chloride injection, uterine artery embolization, and ligation of cervical branches of uterine arteries. The most commonly reported medical treatments are methotrexate, by either local injection (intra-amniotic or intrafetal) or systemic administration [6, 9, 10]. The outcome of conservative management with methotrexate has been reported to be relatively safe and effective, allowing for uterine preservation in more than 90% of cases treated before 12 weeks of gestation [6, 9, 10].

In the case presented, the patient desired to preserve fertility. Inpatient conservative therapy with multidose methotrexate was elected. Although her *β*-hCG levels declined appropriately over the course of her treatment, she re-presented with profuse bleeding per vagina. In the event of hemorrhage following failed medical therapy or postsuction curettage, mechanical compression or tamponade of the cervix can rapidly restore hemostasis [7, 11]. There are several types of balloon catheters that have been described for cervical compression in the literature [12]. These include the Bakri™ Balloon (Cook Medical, Bloomington, Indiana), Rusch hydrostatic catheter (Teleflex Medical Sdn Bhd, Kamuntung, Malaysia), condom catheters, and Foley catheters [12]. Fylstra describes treatment of cervical pregnancy being achieved successfully in 13 patients by curettage immediately followed by placement of 30 mL balloon Foley catheter for tamponade; tamponade was left in place for 24 hours [13]. The use of Foley balloon has also been cited to prophylactically control bleeding in cervical pregnancies [13, 14]. Similarly, Timor-Tritsch successfully treated cervical and cesarean scar ectopic pregnancies with double balloon catheter [15]. The double balloon catheter provided compression to terminate pregnancy and prevent bleeding. Our case report defers from both Fylstra and Timor-Tritsch in that use of double balloon catheter was to control hemorrhage with tamponade, not to prophylactically prevent hemorrhage nor to treat a live ectopic pregnancy.

As an alternative to the Foley catheter, in our patient, tamponade and further hemostasis were created with the Cook Balloon by the insufflation of the internal and external balloon, respectively. Although the Cook Balloon is normally used for cervical ripening for induction of labor, its use has been reported for the management of postabortal hemorrhage [12]. Unlike other compression devices such as the Foley balloon, the Cook catheter consists of a double balloon system. This allows for one balloon to be positioned below the internal-external cervical os and the second balloon to be situated above in the internal cervical os (Figure 1). Tamponade with a double balloon cervical ripening catheter has not been previously described to control hemorrhage in CP. The double balloon additionally adjusts to the length of the cervix prior to insufflation allowing for compression from internal to external os (Figure 2). A double balloon catheter, as used in our case, may be considered to control hemorrhage for CP as it was able to quickly create a tamponade by compressing the cervix from the internal to external os.

Tranexamic acid (TXA) in the case presented was used prophylactically to prevent any further event of hemorrhage. Tranexamic acid is a potent antifibrinolytic agent that exerts its effect by competitively blocking lysine binding sites on plasminogen molecules [16]. This prevents the binding of fibrin to plasminogen and therefore impairs fibrinolysis [16]. The use of TXA in cervical pregnancy has limited discussion in literature. However, the use of TXA in postpartum hemorrhage and trauma is a field of evolving study [17]. Clinically, TXA has been shown to decrease the need of blood transfusions, reoperation for bleeding, and mean of transfused units [17]. TXA's use for menorrhagia has shown to reduce mean blood loss, which may suggest a possible link in reducing uterine blood flow [18].

## 4. Conclusion

The case presented delineates the use of conservative management and complications of cervical pregnancy. The use of methotrexate is well documented in literature for the treatment of cervical pregnancy. However, the use of a double balloon cervical ripening catheter (Cook Balloon) to achieve cervical tamponade during hemorrhage has limited citing in literature. In cases where future fertility is of greatest concern, the Cook Balloon may serve as an option for decreasing blood loss. Furthermore, the use of TXA to prevent future hemorrhage and decrease the need for reoperation is a possible consideration when patient's desire to maintain fertility is a major concern. The prophylactic use of TXA is not well established in cervical pregnancies and future studies are needed to demonstrate its effectiveness.
